# Salt Stress Responses of Different Rice Varieties at Panicle Initiation: Agronomic Traits, Photosynthesis, and Antioxidants

**DOI:** 10.3390/plants14152278

**Published:** 2025-07-24

**Authors:** Yusheng Li, Yuxiang Xue, Zhuangzhuang Guan, Zhenhang Wang, Daijie Hou, Tingcheng Zhao, Xutong Lu, Yucheng Qi, Yanbo Hao, Jinqi Liu, Lin Li, Haider Sultan, Xiayu Guo, Zhiyong Ai, Aibin He

**Affiliations:** 1 School of Breeding and Multiplication (Sanya Institute of Breeding and Multiplication), Hainan University, Sanya 572000, China; yushengli@hainanu.edu.cn (Y.L.); 13625050132@163.com (Y.X.); Guan6187633@163.com (Z.G.); 20203103135@hainanu.edu.cn (Z.W.); 17634021231@163.com (T.Z.); lluuxxutt@163.com (X.L.); 22220951310084@hainanu.edu.cn (Y.Q.); yanbohao2024@163.com (Y.H.); 15850949941@163.com (J.L.); sultanhaider@hainanu.edu.cn (H.S.); 2 National Innovation Center of Saline-Alkali Tolerant Rice in Sanya, Sanya 572000, China; lilin18@hainanu.edu.cn (L.L.); wanilybao@163.com (X.G.); 3 College of Environment and Ecology, Hunan Agricultural University, Changsha 410128, China; houdaijie@163.com; 4 Hunan Hybrid Rice Research Center, Changsha 410125, China

**Keywords:** rice salt tolerance, panicle initiation stage, differential response, agronomic traits, antioxidant levels, physiological adaptation

## Abstract

The utilization of saline–alkali land for rice cultivation is critical for global food security. However, most existing studies on rice salt tolerance focus on the seedling stage, with limited insights into tolerance mechanisms during reproductive growth, particularly at the panicle initiation stage (PI). Leveraging precision salinity-control facilities, this study imposed four salt stress gradients (0, 3, 5, and 7‰) to dissect the differential response mechanisms of six rice varieties (YXYZ: Yuxiangyouzhan, JLY3261: Jingliangyou3261, SLY91: Shuangliangyou91, SLY138: Shuangliangyou138, HLYYHSM: Hualiangyouyuehesimiao, and SLY11:Shuangliangyou111) during PI. The results revealed that increasing salinity significantly reduced tiller number (13.14–68.04%), leaf area index (18.58–57.99%), canopy light interception rate (11.91–44.08%), and net photosynthetic rate (2.63–52.42%) (*p* < 0.001), accompanied by reactive oxygen species (ROS)-induced membrane lipid peroxidation. Integrative analysis of field phenotypic and physiological indices revealed distinct adaptation strategies: JLY3261 rapidly activated antioxidant enzymes under 3‰ salinity, alleviating lipid peroxidation (no significant difference in H_2_O_2_ or malondialdehyde content compared to 0‰ salinity) and maintaining tillering and aboveground biomass. SLY91 tolerated 7‰ salinity via CAT/POD-mediated lipid peroxide degradation, with H_2_O_2_ and malondialdehyde contents increasing initially but decreasing with escalating stress. These findings highlight genotype-specific antioxidant strategies underlying salt-tolerance mechanisms and the critical need for integrating phenomics–physiological assessments at reproductive stages into salt-tolerance breeding pipelines.

## 1. Introduction

Soil salinization poses a significant global challenge to agricultural productivity. Accelerated industrialization, urban expansion, and intensified climate change have led to global rising temperatures, prolonged droughts, and altered precipitation patterns, collectively enhancing surface evaporation and driving continuous salt accumulation in soils [1]. Concurrently, anthropogenic activities, including suboptimal irrigation practices, excessive groundwater extraction, and inadequate coastal protection leading to saltwater intrusion, further exacerbate soil salinity in coastal regions [2,3]. Globally, approximately 1 billion hectares of land are affected by salt stress, with irrigated cropland accounting for 20% of this total [4,5]. Projections indicate that without effective interventions, salinization could threaten 50% of arable land by 2050, severely compromising global food security [6].

Rice (*Oryza sativa* L.), as the staple crop for half the world’s population, is significantly affected by salinity throughout its life cycle, from germination to maturity [7]. Studies demonstrate that mild salinity (electrical conductivity: 5 dS m^−1^) can reduce rice yields by up to 40%, while severe salinity (11 dS m^−1^) may cause losses exceeding 80% [8]. Salt stress adversely affects rice plants through dual mechanisms: osmotic stress and ionic toxicity [9]. Osmotic stress arises from high Na^+^ concentrations in saline soils, which lower rhizospheric water potential, disrupt root water uptake [10], and cause cellular water deficit through reduced turgor pressure. Ionic toxicity results from excessive Na^+^ accumulation in cells, disturbing ion homeostasis, inhibiting enzyme activity, and impairing protein synthesis [11], thereby compromising photosynthetic functions. Leaves, as the primary organs for photosynthesis and transpiration, are key targets of salt stress. To minimize water loss, rice actively closes stomata, reducing intercellular CO_2_ concentration [12] and directly suppressing photosynthetic assimilation efficiency [13,14]. Simultaneously, stress-induced reactive oxygen species (ROS) overproduction triggers lipid peroxidation, damaging chloroplast membrane integrity and grana lamellae structure [15,16]. These alterations accelerate chlorophyll degradation and disrupt photosystem functionality [17]. In addition, differences between rice varieties in terms of ion balance capacity, osmotic regulation capacity, reactive oxygen species scavenging capacity, and cell damage repair capacity ultimately manifest as distinct responses to salt stress [18,19]. Such physiological impairments manifest morphologically as suppressed tillering, reduced leaf area index, diminished canopy light interception, and phenotypic abnormalities, including premature leaf senescence and rolling [20,21]. The synergistic effects of inadequate photoassimilate accumulation and structural damage establish a persistent negative feedback effect on plant growth, ultimately jeopardizing yield formation.

Exploring saline–alkali lands as potential rice cultivation resources is strategically important for expanding arable land, ensuring agricultural sustainability, and safeguarding global food security. Developing salt-tolerant rice varieties constitutes a cost-effective strategy to address salinization challenges. Nevertheless, current salinity-tolerance evaluations predominantly focus on seedling stages or controlled hydroponic environments, while precise field-level salinity control during reproductive growth remains constrained by environmental heterogeneity and geographical limitations [22]. Consequently, research on salt tolerance during reproductive development is significantly lacking. Rice sensitivity exhibits significant variation across different growth stages, and the panicle initiation phase, which marks the onset of reproductive development, exerts a substantial influence on yield. This is attributable to the suppression of growth and panicle fertility that is salinity-mediated [23]. However, existing studies prioritize physiological responses during germination and seedling stages, lacking systematic analyses of photosynthetic regulation (e.g., stomatal conductance and photosynthetic rate dynamics) and antioxidant adaptation (e.g., ROS-scavenging enzyme expression) during the panicle initiation stage [24]. This knowledge gap hinders accurate field-level evaluation of varietal salt tolerance and impedes the establishment of comprehensive, whole-growth-stage salt-tolerance assessment protocols.

Against this backdrop, this study capitalizes on the high-precision salinity-control infrastructure of the National Salt-Tolerant Rice Center to perform multi-gradient salt stress experiments. The research aims to achieve two key objectives: (1) to clarify the varietal disparities in growth characteristics, photosynthetic traits, and antioxidant responses under salt stress during the panicle initiation stage; (2) to fill the research void in field-based salt-tolerance screening at the reproductive stage, thereby furnishing crucial data for formulating whole-growth-stage evaluation criteria and promoting the targeted breeding of salt-tolerant rice varieties.

## 2. Materials and Methods

### 2.1. Experimental Site

The salt pool experiment was carried out in 2025 at the salt–alkali base of the National Innovation Center for Salt-Tolerant Rice Technology in DaDan Village, Yacheng Town, Sanya City, Hainan Province, China (18°36′ N, 109°15′ E). The experimental station is established in a coastal zone equipped with high-precision salinity control and brine distribution systems, enabling precise simulation of natural field salinity gradients. The integrated facility comprises an automated seawater intake network drawing water from estuary-proximal channels and subsurface aquifers, coupled with real-time salinity monitoring arrays in brine storage ponds and mixing chambers. A hierarchical irrigation network distributes treated brine to experimental plots, while automated mixing mechanisms within brine chambers ensure precise solution preparation through dynamic flow regulation. In addition, the station is equipped with a fully automated rain shelter, which is normally closed and will open automatically when the rain sensor senses rainfall, reducing the impact of rainfall on the stable control of salinity. The configuration of this system enabled strict control over salinity parameters during the experiment. Prior to the experiment, double-season rice had been planted in the paddy field for many years. Based on the measurements, the environment between the salt ponds was relatively consistent and stable in this season’s experiment. The soil in the experimental ponds is sandy loam. The pH, total nitrogen (N), available phosphorus, potassium, organic matter, and electrical conductivity in the upper 20 cm of the soil before rice planting were 7.70, 0.71 g kg^−1^, 158.68 mg kg^−1^, 245.24 mg kg^−1^, 8.35 g kg^−1^, and 0.47 dS m^−1^, respectively.

### 2.2. Experimental Design

The proposed study was laid out in a split-zone experimental design with three replications, and the area of each salt plot was 20 m^2^ (4 m × 5 m). The testing rice varieties were allotted to the main plot: a total of six varieties were used in this experiment (Table 1), namely, Yuxiangyouzhan (YXYZ, a conventional rice variety, used as the control variety of the national salinity-tolerant rice district test consortium), Jingliangyou3261 (JLY3261, a high-quality disease-resistant and salt-tolerant hybrid rice, a new variety that passed the national certification in 2025), Shuangliangyou138 (SLY138), Shuangliangyou111 (SLY111), Shuangliangyou91 (SLY91), and Hualiangyouyuehesimiao (HLYYXSM). At the same time, irrigation saline concentrations were allotted to each subplot, including salt concentrations of 0‰ (S0, 0 dS m^−1^, CK), 3‰ (S3, 4.7 dS m^−1^,), 5‰ (S5, 7.8 dS m^−1^), and 7‰ (S7, 10.9 dS m^−1^). After transplanting, irrigation water with different salinity concentrations was continuously applied until harvest, during which salinity monitoring facilities continuously monitored and regulated the salinity stability of the plots. The aerial view of the experimental field is shown in Figure S1.

Rice seeds of six varieties were placed in an oven at 40 °C for 24 h to break dormancy. Then the germinated seeds were sown into seedling trays on 8 February 2025 and manually transplanted into salt ponds on 21 February 2025. All salt ponds were plowed and puddled before transplanting. The density of transplanting was 20 cm × 20 cm, with single seedlings per hill. Plants were fertilized with 100:60:50 kg ha^−1^ of N: P: K. The nitrogen fertilizer was applied as basal fertilizer with tillering fertilizer at the ratio of 1:1. Phosphorus fertilizer was applied once as basal fertilizer. Potassium fertilizer was applied as basal fertilizer. The sources of N, P, and K fertilizers were urea (46.4% N), calcium superphosphate (16.0% P_2_O_5_), and potassium chloride (60.0% K_2_O), respectively. During the experiment, manual weeding was carried out, and according to the early warning of disease and insect disaster issued by the agricultural extension service agencies, combined with actual field observation, we regularly sprayed with pesticides from the middle tillering period of rice and periodically changed the pharmaceutical brand. Specifically, insecticides such as Abamectin (1.8% emulsifiable concentrate, 150 mL/ha) and Imidacloprid (10% wettable powder, 300 g/ha) were used to control rice planthoppers and stem borers. Fungicides such as Carbendazim (50% wettable powder, 750 g/ha) and Difenoconazole (10% emulsion, 200 mL/ha) were applied to prevent rice blast and stem rot. All dosages were calibrated according to the manufacturer’s instructions for tropical rice cultivation in Hainan, ensuring compliance with safety standards.

The panicle initiation stage (PI) was defined as the developmental stage around 25 days after transplanting, characterized by the panicle primordia length of approximately 2.0 mm observed under a stereomicroscope (SZX16; Olympus Corporation, Japan) with distinct differentiation of primary branches (visible as protuberances on the rachis).

The flowchart for this experiment is shown in Supplementary Figure S2.

### 2.3. Determination of Tiller Number, Plant Height, and Aboveground Dry Weight (ADW)

At the PI, 6 plants (0.24 m^2^) were randomly taken for growth analysis in each plot. The plants were washed, and the tiller number and plant height were recorded. After removing the roots with scissors, the plants were divided into culms and leaves. The samples were dried in an oven at 105 °C for half an hour and then at 80 °C for 2 days until they reached a constant weight for weighing the ADW.

### 2.4. Canopy Interception Rate (CIR) and Leaf Area Index (LAI)

At the PI of rice, the cumulative incident light and light interception of the canopy were measured via a canopy analyzer (AccuPAR LP-80; Decagon Devices Inc., Pullman, WA, USA) to calculate the light interception rate (LI%) according to [25]. Four representative and uniformly growing rice plants were selected from each replicate, the whole intact green leaves were removed, the leaf area was measured and recorded using a hand-held laser leaf area meter (CI-203; Shanghai Zequan Science and Technology Co., Ltd., CID, Shanghai, China), and the LAI was calculated.

### 2.5. Leaf SPAD Value

The SPAD value was measured at the PI using a portable chlorophyll meter (SPAD-502 PLUS; Konica Minolta, Tokyo, Japan). Four uniform plants were selected from each replicate; sword leaves were measured, avoiding the leaf veins, with three measurements taken on the upper, middle, and lower parts of each leaf; and the average value was taken.

### 2.6. Parameters of Photosynthetic Characteristics

At the PI, three uniform plants were selected from each replicate, and the uppermost rice leaves were placed in the fluorescent leaf chamber (6800-01A) of the portable photosynthesis tester (LI-6800F, LI-COR, Lincoln, NE, USA). The saturating light intensity was set at 1800 μmol m^−2^ s^−1^, and the CO_2_ concentration in the leaf chamber was set at 400 μmol mol^−1^. The net photosynthetic rate (Pn), transpiration rate (Tr), stomatal conductance (Gs), and intercellular CO_2_ concentration (Ci) of leaves under saturated light were recorded.

### 2.7. Determination of Activities of Antioxidant Enzymes, H_2_O_2_ Content, and Malondialdehyde (MDA) Content

At the PI, the uppermost rice leaf samples from each treatment were cut, immersed in liquid N_2_, and stored at −80 °C in an ultra-low-temperature freezer. Antioxidant enzymes, including superoxide dismutase (SOD), peroxidase (POD), and catalase (CAT), and H_2_O_2_ content and MDA content were determined according to the instructions for the kits produced by Beijing SoleiBao Technology Co., Ltd., Beijing, China. Refer to [26] for detailed test methods.

### 2.8. Data Analysis

Data were analyzed through Microsoft Excel 2016 (Microsoft Inc., Redmond, WA, USA) and analysis of variance (ANOVA) using Statistix 9.0 software (Analytical Software, Tallahassee, FL, USA). The differences between treatments were separated using the least significance difference (LSD) test at the 0.05, 0.01, and 0.001 probability levels. All graphs were created using Origin 9.0 (OriginLab Corp., Northampton, MA, USA).

## 3. Results

### 3.1. Plant Height, Tiller Numbers, and Aboveground Dry Weight

**Figure 1:**
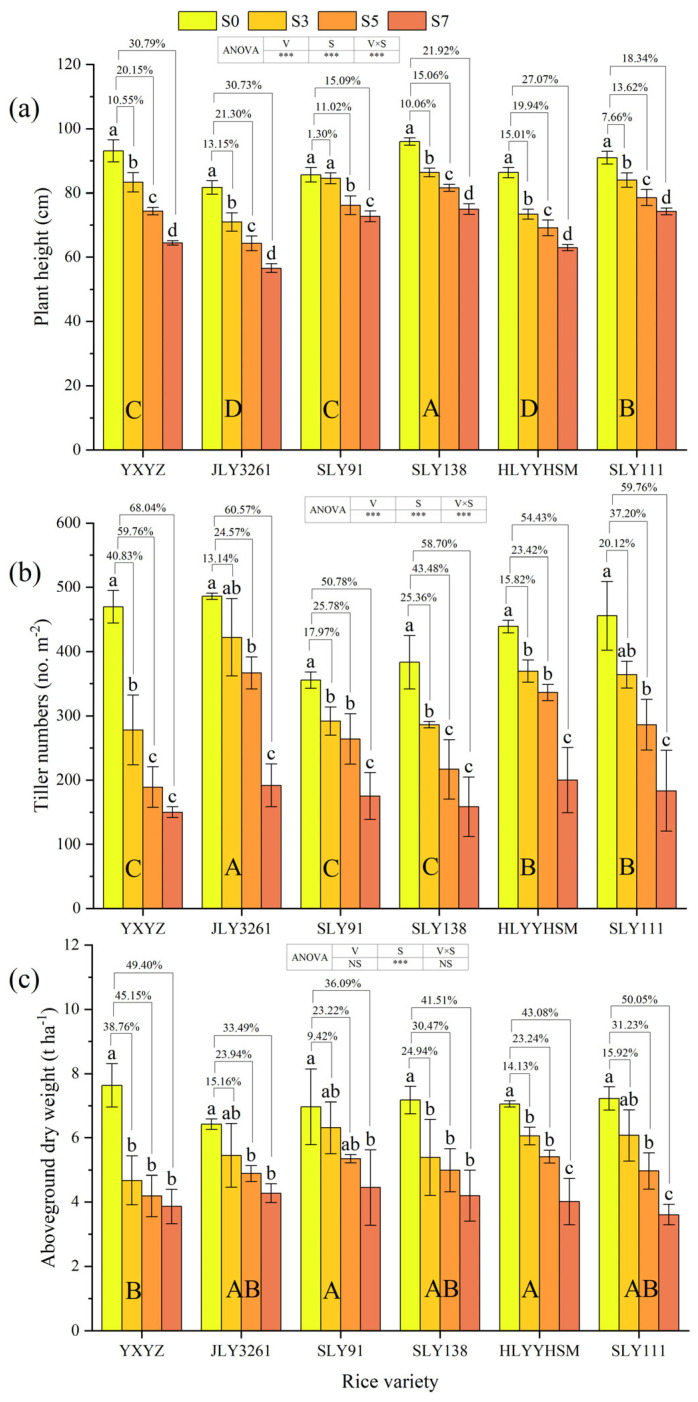
Effects of different rice varieties and different degrees of salt stress on (**a**) plant height, (**b**) tiller numbers, and (**c**) aboveground dry weight at panicle initiation stage (see Section 3.1). Note: YXYZ represents Yuxiangyouzhan, JLY3261 represents Jingliangyou3261, SLY91 represents Shuangliangyou91, SLY138 represents Shuangliangyou138, HLYYHSM represents Hualiangyouyuehesimiao, and SLY111 represents Shuangliangyou91. S0, S3, S5, and S7 represent the 0, 3, 5, and 7‰ saline levels, respectively. Data are means ± SDs. The values indicated by the connecting lines above the columns are the rates of changes between rice treated with different salt stresses (S3, S5, and S7) and CK (S0). Different uppercase letters in the columns indicate significant differences among rice varieties across the four salinity gradients (*p* ≤ 0.05, LSD Test). Different lowercase letters in the columns indicate significant differences in the same rice variety under different degrees of salt stress at the 0.05 level (*p* ≤ 0.05) according to the LSD test. In the ANOVA analysis table, V represents variety, S represents salinity, and V × S represents the interaction of variety and salinity. * indicates an interaction at the 0.05 level (*p* ≤ 0.05), ** indicates an interaction at the 0.01 level (*p* ≤ 0.01), *** indicates an interaction at the 0.001 level (*p* ≤ 0.001), and NS indicates that there is no interaction.

Plant height and tiller numbers were significantly affected by variety, salinity, and their interaction (*p* < 0.001). Salinity alone strongly influenced aboveground dry weight (*p* < 0.001), while variety and its interaction with salinity did not (Figure 1a–c). All varieties showed progressive declines in plant height with increasing salinity: SLY138 remained tallest, JLY3261 the shortest across treatments. SLY91 had the lowest growth inhibition (9.14%), with no significant difference between S3 and CK; S7 most severely inhibited YXYZ and JLY3261. Tiller numbers decreased with rising salinity: JLY3261 produced the most, SLY138 the fewest. JLY3261 and SLY111 showed no significant tiller differences between S3 and CK. Aboveground dry weight declined gradually with salinity: SLY91 accumulated the most biomass, YXYZ the least. JLY3261 and SLY91 were least suppressed (24.20% and 22.91% inhibition), with S3 plants of JLY3261, SLY91, and SLY111 showing no significant difference from CK. S7 most severely impacted YXYZ and SLY111.

### 3.2. Canopy Interception Rate and Leaf Area Index

**Figure 2:**
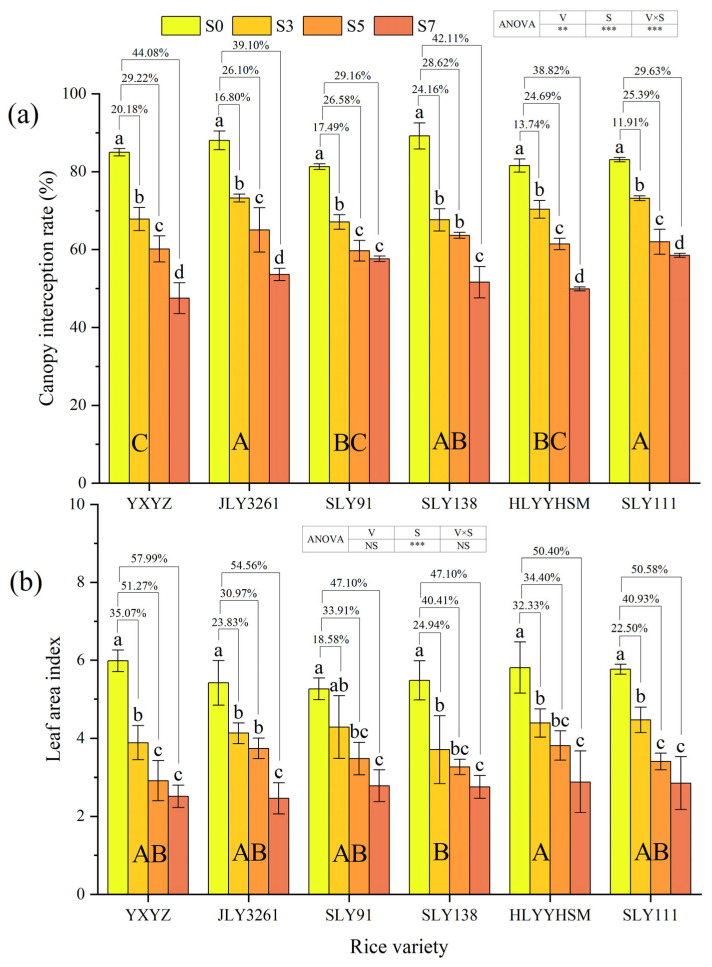
Effects of different rice varieties and different degrees of salt stress on (**a**) canopy interception rate and (**b**) leaf area index at panicle initiation stage (see Section 3.2). Note: Variety abbreviations, salt stress gradients, statistical annotations, and data calculation rules follow Figure 1.

Rice variety (*p* < 0.01), salinity, and their interaction (*p* < 0.001) significantly influenced canopy interception rate (Figure 2a). Progressive reductions in canopy interception rates were observed across all cultivars with increasing salt concentrations, where JLY3261 and SLY111 maintained higher interception capacity under salt stress, while YXYZ showed the lowest value. During the panicle initiation stage, canopy interception rate reductions relative to CK under the S3, S5, and S7 treatments were quantified as 20.18, 29.22, and 44.08% for YXYZ; 16.80, 26.10, and 39.10% for JLY3261; 17.49, 26.58, and 29.16% for SLY91; 24.16, 28.62, and 42.11% for SLY138; 13.74, 24.69, and 38.82% for HLYYHSM; and 11.91, 25.39, and 29.63% for SLY111. Notably, YXYZ and SLY138 experienced the most pronounced salt-induced suppression, with overall inhibition rates of 31.16% and 31.63%, respectively. In contrast, SLY111 showed the most mitigated impact (22.31% overall inhibition).

Salt stress intensity exerted a highly significant effect on leaf area index (LAI) (*p* < 0.001), whereas neither cultivar nor its interaction with salt showed a significant impact (Figure 2b). A concentration-dependent decline in LAI was observed across all rice varieties under varying salt treatments. At the panicle initiation stage, reductions in LAI under the S3, S5, and S7 treatments relative to the CK were measured as follows: 35.07, 51.27, and 57.99% for YXYZ; 23.83, 30.97, and 54.56% for JLY3261; 18.58, 33.91, and 47.10% for SLY91; 24.94, 40.41, and 47.10% for SLY138; 32.33, 34.40, and 50.40% for HLYYHSM; and 22.50, 40.93, and 50.58% for SLY111. Notably, YXYZ exhibited the most severe salt-induced suppression (overall inhibition rate: 48.11%), while SLY91 demonstrated relatively minor impairment (33.20%). Intriguingly, SLY91 under S3 treatment showed no significant LAI difference compared to controls, suggesting partial stress tolerance at lower salinity.

### 3.3. Leaf SPAD Value

**Figure 3:**
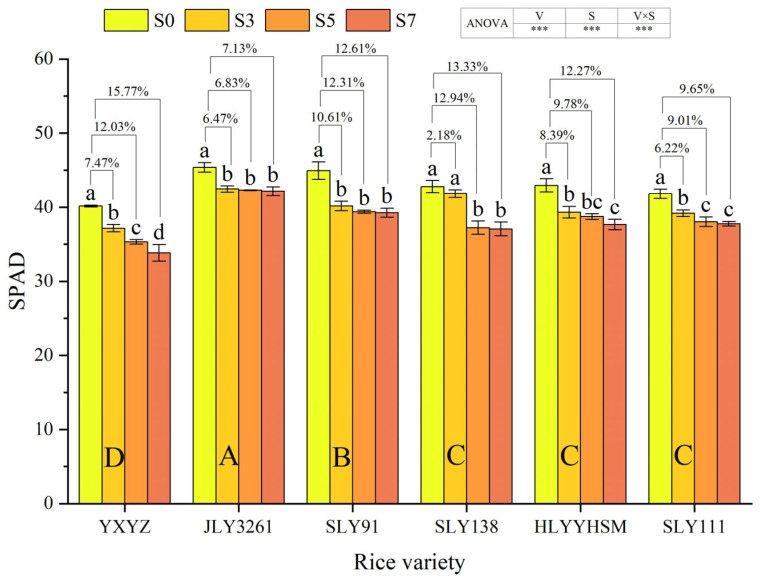
Effects of different rice varieties and different degrees of salt stress on leaf SPAD at panicle initiation stage (see Section 3.3). Note: Variety abbreviations, salt stress gradients, statistical annotations, and data calculation rules follow Figure 1.

The rice variety, salinity, and their interaction exerted highly significant effects on leaf SPAD values (*p* < 0.001) (Figure 3). With increasing salt concentration, all tested rice varieties exhibited a declining trend in SPAD values. Among the cultivars, JLY3261 maintained the highest SPAD values across different salt treatments, while YXYZ showed the lowest values. At the panicle initiation stage, compared to CK, YXYZ demonstrated SPAD value reductions of 7.47%, 12.03%, and 15.77% under S3, S5, and S7, respectively. The corresponding reductions for other varieties were 6.47%, 6.83%, and 7.13% for JLY3261; 10.61%, 12.31%, and 12.61% for SLY91; 2.18%, 12.94%, and 13.33% for SLY138; 8.39%, 9.78%, and 12.27% for HLYYHSM; and 6.22%, 9.01%, and 9.65% for SLY111. Salt stress exhibited the strongest inhibitory effect on YXYZ (overall inhibition rate: 11.76%), while JLY3261 showed the least susceptibility to salt-induced SPAD value reduction (overall inhibition rate: 6.81%).

### 3.4. Parameters of Photosynthetic Characteristics

**Figure 4:**
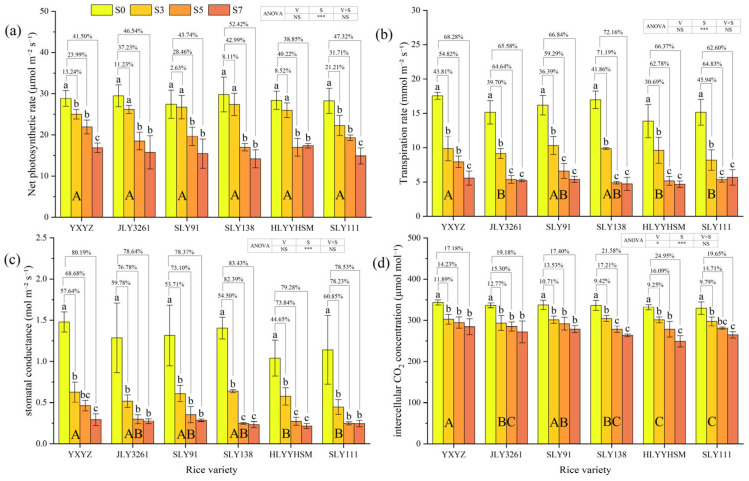
Effects of different rice varieties and different degrees of salt stress on (**a**) net photosynthetic rate, (**b**) transpiration rate, (**c**) stomatal conductance, and (**d**) intercellular CO_2_ concentration at panicle initiation stage (see Section 3.4). Note: Variety abbreviations, salt stress gradients, statistical annotations, and data calculation rules follow Figure 1.

At the panicle initiation stage of rice, salinity exerted highly significant effects (*p* < 0.001) on following photosynthetic parameters: net photosynthetic rate (Pn), transpiration rate (Tr), stomatal conductance (Gs), and intercellular CO_2_ concentration (Ci), while there were also differences in responses among varieties (Figure 4a–d). Compared to CK, Pn decreased by 2.63% to 52.42%. Notably, JLY3261, SLY91, SLY138, and HLYYHSM under the S3 treatment showed no significant difference in Pn compared to CK. Salt stress significantly reduced Tr, Gs, and Ci across cultivars, with decreases of 30.69–72.16%, 44.64–83.43%, and 9.25–24.95%, respectively. Under low salt stress (S3), all three parameters were significantly lower than CK. At higher salt levels (S5 and S7), these parameters were highly significantly reduced, with minimal differences observed between S5 and S7.

### 3.5. Activities of Antioxidant Enzymes, H_2_O_2_ Content, and MDA Content

**Figure 5:**
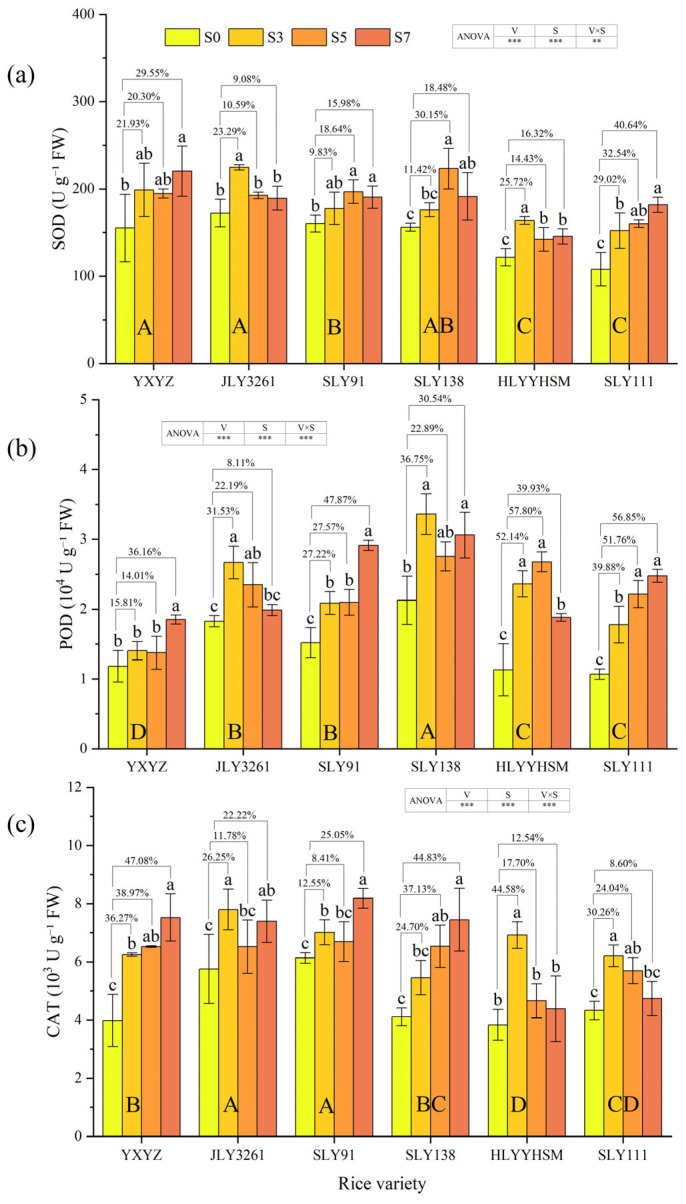
Effects of different rice varieties and different degrees of salt stress on (**a**) SOD activity, (**b**) POD activity, and (**c**) CAT activity at panicle initiation stage (see Section 3.6). Note: Variety abbreviations, salt stress gradients, statistical annotations, and data calculation rules follow Figure 1.

The activities of three antioxidant enzymes (SOD, POD, and CAT) significantly (*p* < 0.001) increased under salt stress compared to CK, and the responses of enzyme activities to varying salt stress levels differed among rice cultivars (Figure 5a–c). With the increase in salt concentration, the activities of the three enzymes in JLY3261 first increased and then decreased, i.e., S3 > S5/S7 > S0, and under 3‰ salt stress, the activities of SOD and CAT in JLY3261 were higher than those in the other varieties. The activities of the three enzymes in SLY91 increased with the increase in salt stress. It is worth noting that under 7‰ salt stress, the activities of POD and CAT in SLY91 increased by 47.87% and 25.05%, respectively, compared with CK, and the activity of CAT was higher than that in other varieties.

**Figure 6:**
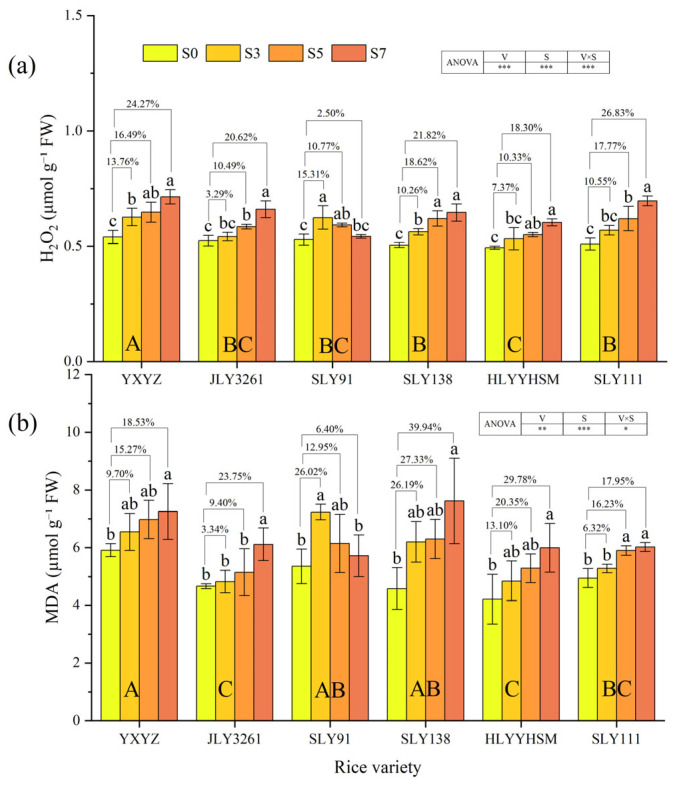
Effects of different rice varieties and different degrees of salt stress on (**a**) H_2_O_2_ content and (**b**) MDA content at panicle initiation stage (see Section 3.6). Note: Variety abbreviations, salt stress gradients, statistical annotations, and data calculation rules follow Figure 1.

Both rice varieties and salinity had a highly significant effect on the H_2_O_2_ and MDA contents in rice leaves (*p* < 0.01) (Figure 6a,b). For H_2_O_2_ content, all varieties showed dynamic changes with escalating salt stress, but the patterns varied distinctly by genotype. YXYZ exhibited the most severe accumulation. In contrast, JLY3261 maintained relatively stable H_2_O_2_ levels, with only a 3.29% increase at S3 and no significant difference from CK (*p* > 0.05), indicating efficient early ROS scavenging. Notably, SLY91 displayed a unique “first rise, then fall” trend: H_2_O_2_ content increased by 15.31% at S3 but decreased to 2.50% above CK at S7, suggesting enhanced ROS degradation capacity under high salinity. YXYZ exhibited the highest MDA content under various salt treatments. With the increase in salt concentration, the MDA content increase rates of different varieties compared to CK were as follows: YXYZ: S7 (18.53%) > S5 (15.27%) > S3 (9.70%); JLY3261: S7 (20.75%) > S5 (9.40%) > S3 (3.34%); SLY91: S7 (6.40%) < S5 (12.95%) < S3 (26.02%); SLY138: S7 (39.94%) > S5 (27.33%) > S3 (26.19%); HLYYHSM: S7 (29.78%) > S5 (20.35%) > S3 (13.10%); SLY111: S7 (17.95%) > S5 (16.23%) > S3 (6.32%). The MDA content of most rice varieties increased with higher salinity, while SLY91 showed a trend of first increasing and then decreasing. Notably, there was no significant difference in MDA content between SLY91 under S7 salt stress and the CK. JLY3261 had the smallest overall increase in MDA content (12.17%), with no significant differences from the CK under S3 and S5 salt stress.

### 3.6. Correlation Analysis of Agronomic Traits, Photosynthetic Indices, and Antioxidant Capacity in Rice

**Figure 7:**
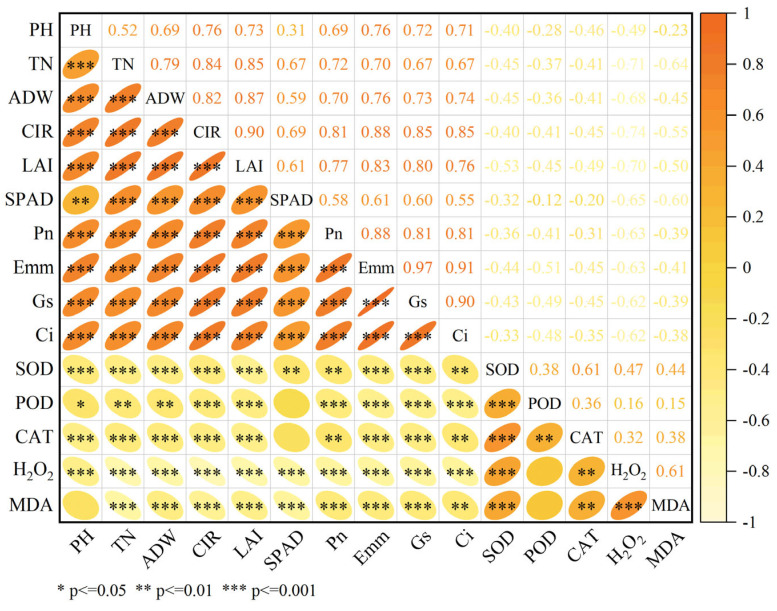
Correlation analysis of agronomic traits, photosynthetic indices, and antioxidant capacity in rice (*n* = 72). The heatmap displays Pearson correlation coefficients, with colors ranging from yellow (−1) to red (+1). Rows and columns represent agronomic traits of rice (PH = plant height, TN = tiller number, ADW = aboveground dry weight, CIR = canopy interception rate, LAI = leaf area index, SPAD = SPAD value, Pn = net photosynthetic rate, Tr = transpiration rate, Gs = stomatal conductance, Ci = intercellular carbon dioxide concentration, SOD = SOD enzyme activity, POD = POD enzyme activity, CAT = CAT enzyme activity, H_2_O_2_ = H_2_O_2_ content, MDA = MDA content). Data are based on *n* = 3 biological replicates per treatment (6 rice varieties × 4 salinity levels). Asterisks (*) denote significance levels: * *p* < 0.05, ** *p* < 0.01, *** *p* < 0.001.

Correlation analysis revealed complex interrelationships among agronomic traits, photosynthetic parameters, and antioxidant levels (Figure 7). Significant positive correlations (r > 0.5, *p* < 0.001) were observed between all measured agronomic traits: plant height, tiller number, aboveground dry weight, canopy interception rate, and leaf area index. Regarding photosynthetic parameters, net photosynthetic rate (Pn) demonstrated significant positive correlations with SPAD value (r = 0.58; *p* < 0.001), transpiration rate (Tr, r = 0.88; *p* < 0.001), stomatal conductance (Gs, r = 0.81; *p* < 0.001), and intercellular CO_2_ concentration (Ci, r = 0.81; *p* < 0.001). Among antioxidant enzymes, synergistic interactions were evidenced by significant positive correlations between SOD, POD, and CAT activities (*p* < 0.01 for all pairwise comparisons). A significant positive correlation between H_2_O_2_ and MDA (*p* < 0.001) provided evidence of their synergistic interactions. Additionally, H_2_O_2_ and MDA contents exhibited positive correlations with antioxidant enzyme activities, reaching statistical significance for SOD (*p* < 0.001) and CAT (*p* < 0.01), though their correlation with POD was non-significant (NS). Notably, agronomic traits showed positive correlations with photosynthetic parameters, whereas both agronomic traits and photosynthetic parameters were negatively correlated with antioxidant levels.

## 4. Discussion

### 4.1. Differential Effects of Salt Stress on Agronomic Traits

Salt stress, a pivotal abiotic stressor, exerts significant inhibitory effects on multiple agronomic traits in rice, including plant height, tiller number, and aboveground dry weight (*p* < 0.001), with the degree of inhibition escalating in tandem with salt concentration. ANOVA revealed significant genotypic differences in plant height and tiller number (*p* < 0.001) (Figure 1). Plant height, a core morphological indicator of crop response to stress, and its inhibitory mechanism is closely related to impaired cell elongation, water absorption disorders, and altered distribution of photosynthetic products [27]. In the present study, SLY91 exhibited a 15.09% reduction in plant height under high salinity (S7), the lowest among all varieties, and only a 1.3% reduction under low salinity (S3), showing no significant difference from CK (Figure 1a). This phenomenon was likely attributed to the ability of salt-tolerant varieties to maintain cell turgor and cell wall elasticity, thereby mitigating the inhibition of stem elongation under salt stress [28]. In contrast, YXYZ exhibited a 30.79% reduction in plant height under high salinity (S7), reflecting differences in cellular structural sensitivity to salt stress.

Tillering number, a key yield-component trait, has been shown in previous studies to be a primary factor contributing to salt-induced yield loss, as reduced tiller survival leads to diminished panicle formation [23]. Notable disparities in tillering inhibition rates were observed among different varieties. JLY3261 and SLY111 showed no significant differences in tillering numbers compared to the control under S3, while YXYZ exhibited a high tillering inhibition rate of 40.83% (Figure 1b). This difference is closely related to the inhibition of tiller bud initiation and development. Studies have shown that salt stress suppresses the cellular activity of tiller primordia by disrupting endogenous hormone balance (e.g., ABA accumulation) and ion homeostasis (e.g., K^+^/Na^+^ imbalance) [21,29]. Salt-tolerant varieties protect tiller primordia from ionic toxicity by maintaining lower Na^+^ accumulation and higher K^+^/Na^+^ ratios, thereby retaining more effective tillers and laying the foundation for panicle number [30]. Notably, the strong correlation between tiller number and yield components (e.g., panicle formation rate) establishes it as a core phenotypic marker distinguishing salt-tolerant from salt-sensitive varieties.

Fluctuations in aboveground dry weight serve as a reflection of overall plant growth, with the panicle initiation stage representing a critical transition from vegetative to reproductive growth. Salt-tolerant varieties maintain higher aboveground biomass (stems and leaves) under salt stress, providing abundant photosynthates and energy for young panicle (sink) development through robust source tissues [8]. Correlation analysis showed significant positive relationships between plant height, tiller number, and aboveground dry weight during panicle initiation (*p* < 0.001) (Figure 7), revealing a cascading inhibition effect of salt stress on rice growth: reduced plant height and tiller number disrupt population structure, hinder aboveground assimilate accumulation and transport, and may further inhibit panicle development and delay heading, ultimately leading to yield loss.

### 4.2. The Response Mechanism of Photosynthetic Capacity to Salt Stress

Photosynthesis, as the core physiological process for biomass accumulation in rice, directly determines assimilate production and yield formation [31]. Previous studies have shown that salt stress inhibits the photosynthetic process by altering leaf morphology, reducing or closing stomata, and decreasing chlorophyll content and Rubisco activity [32]. In this study, canopy interception rate and leaf area index decreased significantly with increasing salt concentration (*p* < 0.001), with YXYZ showing reductions of 44.08% and 57.99% in these parameters under S7 compared to CK (Figure 2). This indicates that salt stress directly weakens canopy light interception by inhibiting tiller formation (Figure 1b) and leaf expansion, thereby reducing the quantity and spatial distribution of photosynthetic organs. Additionally, chlorophyll content, a critical determinant of light absorption efficiency, was monitored using SPAD values, which correlate closely with chlorophyll a/b ratios. SPAD values decreased progressively with increasing salinity (Figure 3), reflecting leaf chlorosis and premature senescence under salt stress. Mechanistically, salinity induces reactive oxygen species (ROS)-mediated membrane lipid peroxidation, disrupting the structural integrity of chloroplast grana lamellae and leading to chlorophyll degradation [33], which represents structural damage to photosynthetic organs [34]. Notable varietal differences were observed: YXYZ exhibited an average SPAD reduction of 11.76%, whereas JLY3261 showed only a 6.81% decrease. This disparity suggests that relatively salt-tolerant varieties maintain chloroplast membrane integrity and slow chlorophyll degradation through a combination of osmotic adjustment for intracellular ion homeostasis and higher antioxidant enzyme activities. Stomatal limitation is another key pathway through which salinity reduces photosynthetic efficiency. Osmotic stress decreases cellular turgor pressure, prompting stomatal closure and subsequent reductions in CO_2_ uptake [35,36]. In this study, salinity significantly reduced transpiration rate (Tr), stomatal conductance (Gs), and intercellular CO_2_ concentration (Ci) (*p* < 0.001), all of which were positively correlated with net photosynthetic rate (Pn) (Figure 4 and Figure 7). However, minimal differences in photosynthetic parameters were observed among S3–S7, implying that Gs had already reached a critically low level under high salinity, becoming the primary limiting factor for photosynthesis. The non-significant varietal differences in gas exchange parameters (except Ci) suggest a universal functional inhibition of the photosynthetic apparatus by salinity, with salt-tolerant varieties relying more on maintaining morphological structures (e.g., tiller number and leaf area) to ensure assimilate accumulation (Figure 1b and Figure 2b). In summary, salt stress inhibits rice photosynthetic capacity through both structural impairments (reduced leaf area and chlorophyll degradation) and functional limitations (stomatal closure and decreased CO_2_ assimilation). Salt-tolerant varieties partially counteract these effects by preserving the quantity and structural integrity of photosynthetic organs, highlighting the importance of integrating morphological and physiological traits in assessing salinity tolerance during the panicle initiation stage.

### 4.3. Effect of Salt Stress on Antioxidant Levels in Different Salt Varieties

Salt stress-induced ion toxicity disrupts electron transport chains (ETCs) in mitochondria and chloroplasts, leading to excessive accumulation of reactive oxygen species (ROS), such as superoxide anions (O_2_^−^) and hydrogen peroxide (H_2_O_2_) [37,38]. Among these, H_2_O_2_ is a relatively stable ROS that can diffuse across membranes and act as a signaling molecule or cause oxidative damage by reacting with cellular components, making its dynamics critical for understanding oxidative stress responses [37]. MDA, a product of lipid peroxidation caused by ROS (particularly H_2_O_2_) attacking polyunsaturated fatty acids in membrane lipids, serves as a marker for oxidative damage, with its accumulation directly reflecting the degree of membrane injury [17]. Notably, our results revealed a consistent trend between H_2_O_2_ content and MDA levels across genotypes (Figure 6a,b), confirming that H_2_O_2_ accumulation is a key driver of membrane lipid peroxidation under salt stress. In this study, MDA content in rice during the panicle initiation stage exhibited a significant upward trend with increasing salt stress (Figure 6a), while the magnitude of MDA increases varied among genotypes, indicating genotype-specific differences in membrane lipid peroxidation levels under salinity [39]. Parallel changes in H_2_O_2_ content further clarified these differences: varieties with severe H_2_O_2_ accumulation (e.g., YXYZ and SLY138) showed higher MDA levels, whereas those with stable or declining H_2_O_2_ (e.g., JLY3261 and SLY91) maintained lower MDA levels, highlighting the causal link between H_2_O_2_ buildup and membrane damage. Salt-tolerant varieties mitigated ROS damage through activation of the enzymatic antioxidant system, with synergistic functions of superoxide dismutase (SOD), peroxidase (POD), and catalase (CAT) being critical. SOD converts O_2_^−^ to H_2_O_2_, while CAT and POD directly scavenge H_2_O_2_—their coordinated activity is therefore essential for preventing H_2_O_2_-mediated oxidative cascades. Antioxidant enzymes maintained the turnover efficiency of the D1 protein in the PSII reaction center by removing ROS (especially H_2_O_2_) near the thylakoid membrane, thereby ensuring the continuity of the photosystem electron transport chain [40]. Antioxidant enzyme activities in all tested varieties increased with escalating salt stress (Figure 5), yet their response patterns differed significantly among genotypes, which was mirrored in H_2_O_2_ dynamics. JLY3261 showed peak activities of SOD, POD, and CAT under low salinity (S3), followed by a gradual decline at higher salt concentrations, suggesting a rapid response to mild stress but potential impairment in enzyme synthesis or activity maintenance under prolonged high salinity. This was corroborated by both H_2_O_2_ and MDA contents in JLY3261: H_2_O_2_ levels remained stable at S3 (only a 3.29% increase vs. CK), with no significant difference in MDA among S0–S5, indicating that efficient early scavenging of H_2_O_2_ prevented membrane lipid peroxidation (Figure 6), thereby maintaining the stability of tiller number and LAI. In contrast, SLY91 exhibited notable enhancement of CAT and POD activities under high salinity (S7) (Figure 5a,b), accompanied by a “first increase, then decrease” trend in both H_2_O_2_ and MDA content (Figure 6a,b). This suggests that SLY91 blocks ROS-mediated lipid peroxidation cascades by intensifying H_2_O_2_ scavenging (dominated by CAT) and lipid peroxide degradation (assisted by POD) [15]. SLY91 may degrade lipid peroxides that have already been generated, reducing MDA content by 7.53% compared to S5 (Figure 6) and ultimately maintaining its higher plant height, leaf area index, and photosynthetic capacity under S7. Notably, although YXYZ showed substantial increases in CAT activity under salt stress, its H_2_O_2_ content exhibited the most severe accumulation (consistent with its highest MDA levels among all varieties), indicating that elevation of a single enzyme activity is insufficient to combat excessive H_2_O_2_ buildup and highlighting the need for coordinated antioxidant enzyme activation to mitigate oxidative damage. Additionally, studies have suggested that Osmo protectants such as proline and betaine may indirectly protect membrane lipids by maintaining osmotic balance [41]. Although these substances were not directly measured in this study, the unique trends in H_2_O_2_ and MDA in SLY91 (increasing at S3, decreasing at S7) imply potential involvement of multiple mechanisms, including enzymatic scavenging combined with osmotic adjustment. In contrast, the antioxidant response of JLY3261 relied more on rapid activation of early stress signaling to control H_2_O_2_ accumulation. These genotype-specific antioxidant strategies, rooted in genetic background-driven stress adaptation, provide physiological indicators for targeted screening of salt-tolerant rice varieties.

This study focuses on the phenotypic and physiological responses (growth traits, photosynthetic characteristics, antioxidant enzymes, ROS, and MDA) of rice varieties during the panicle initiation stage under salt stress. However, it lacks an in-depth exploration of potential molecular mechanisms, such as transcriptomic features (e.g., differential expression of antioxidant-related genes) and metabolomic changes (e.g., variations in non-enzymatic antioxidants or signaling molecules), which limits a comprehensive understanding of the genetic regulatory networks underlying the genotype-specific antioxidant strategies observed. In future research, we will integrate transcriptomic and metabolomic analyses of key genotypes (JLY3261 and SLY91) under salt stress to identify core genes and metabolic pathways involved in their unique ROS scavenging strategies, validate the functions of key regulatory factors, and further develop molecular markers associated with observed physiological traits. This will further promote the integration of phenotypic screening, physiological mechanisms, and molecular breeding in salt-tolerance research.

## 5. Conclusions

This study elucidated the salinity-tolerance mechanisms in rice during panicle initiation. As salinity increased, salt stress inhibited tillering and leaf expansion, disrupted photosynthetic source organs, induced ROS accumulation, and thereby caused membrane lipid peroxidation, reduced photosynthetic efficiency, and decreased biomass. Divergent adaptive strategies were observed among genotypes: JLY3261 rapidly activated SOD/CAT under low salinity, efficiently scavenging ROS to maintain stable MDA levels, tillering, and biomass—reflecting robust early stress responses. SLY91, conversely, enhanced CAT/POD activities under high salinity, with H_2_O_2_ and MDA showing a “first rise, then fall” trend, indicating sustained ROS-scavenging capacity that preserved membrane homeostasis. These findings highlight genotype-specific antioxidant strategies (rapid low-salt activation vs. sustained high-salt upregulation) and their roles in maintaining source–sink balance, providing insights for integrating reproductive-stage phenomics–physiology into salt-tolerance breeding. Future work will explore transcriptomic/metabolomic networks in tolerant cultivars to advance precision breeding.

## Figures and Tables

**Table 1:** The brief description of rice varieties for testing.

Variety	Brief Name	Rice Type	Rice Parentage	Incubation Unit	Trait
Yuxiangyouzhan	YXYZ	Indica conventional rice	TY36/IR100 (♀) × IR100 (♂)	Rice Research Institute of Guangdong Academy of Agricultural Sciences	The control variety of the national salt-tolerant rice district test consortium, with a salt resistance lower than JLY3261
Jingliangyou3261	JLY3261	Indica two-line hybrid rice	Jing196S (♀) × R3261 (♂)	Hunan Hybrid Rice Research Center; National Salt-Tolerant Rice Technology Innovation Center	High-quality disease-resistant and salt-tolerant hybrid rice
Shuangliangyou138	SLY138	Indica two-line hybrid rice	Shuang1S (♀) × Qianghui38 (♂)	Xike Agricultural Group Co., Ltd.; Hunan Hybrid Rice Research Center; Hunan Academy of Agricultural Sciences	High yield, but salt tolerance has not been reported
Shuangliangyou111	SLY111	Indica two-line hybrid rice	Shuang1S (♀) × Shuanghui11 (♂)	Xike Agricultural Group Co., Ltd.; Hunan Hybrid Rice Research Center; Hunan Academy of Agricultural Sciences	Not tolerant to rice blast, salt tolerance not reported
Shuangliangyou91	SLY91	Indica two-line hybrid rice	Shuang1S (♀) × Z91 (♂)	Hunan Hybrid Rice Research Center	/
Hualiangyouyuexiangsimiao	HLYYXSM	Indica two-line hybrid rice	Hua9S (♀) × Yuehesimiao (♂)	National Salt-Tolerant Rice Technology Innovation Center in Sanya; Hunan Hybrid Rice Research Center	/

Note: ‘/’ means the information has not been reported or is unknown.

## Data Availability

The dataset is available on request from the authors.

## Supplementary Materials

The following supporting information can be downloaded at: https://www.mdpi.com/article/10.3390/plants14152278/s1, Figure S1: Aerial view of the experimental field; Figure S2: Experimental design flow chart.
