# Supplementary material for: Salt Stress Responses of Different Rice Varieties at Panicle Initiation: Agronomic Traits, Photosynthesis, and Antioxidants
Source: Plants (Basel). 2025 Jul 24;14(15):2278. doi: 10.3390/plants14152278 (PMC12348872; doi:10.3390/plants14152278)
Supplement: Supplementary file 1 [file plants-14-02278-s001.zip › plants-3771624-supplementary.pdf]

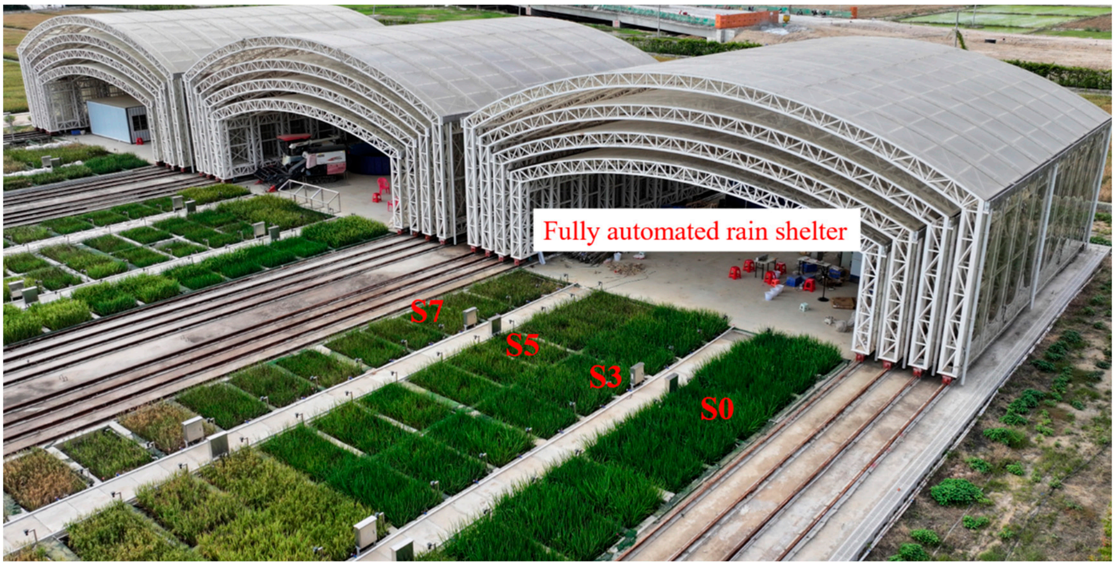

Figure S1. Aerial view of the experimental field. Note: S0, S3, S5 and S7 represent the 0, 3, 5 and 7‰ saline, respectively. The white structure is a fully automatic rain shelter, which remains closed under normal conditions. When the rain sensor detects rainfall, it automatically opens to minimize the impact of rainfall on salinity stability control.

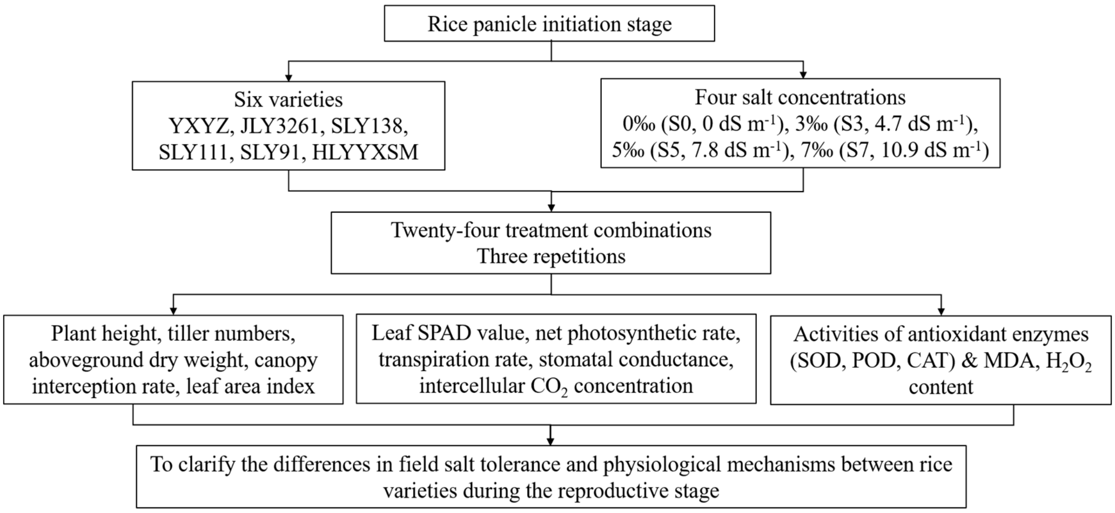

Figure S2. Experimental design flow chart.
